# UAV Assisted Livestock Distribution Monitoring and Quantification: A Low-Cost and High-Precision Solution

**DOI:** 10.3390/ani13193069

**Published:** 2023-09-29

**Authors:** Wenxiang Ji, Yifei Luo, Yafang Liao, Wenjun Wu, Xinyi Wei, Yudie Yang, Xiong Zhao He, Yutong Shen, Qingshan Ma, Shuhua Yi, Yi Sun

**Affiliations:** 1 Institute of Fragile Eco-Environment, School of Geographic Science, Nantong University, 9 Seyuan Road, Nantong 226019, Chinayis@ntu.edu.cn (S.Y.); 2 School of Agriculture and Environment, College of Science, Massey University, Private Bag 11222, Palmerston North 4442, New Zealand; 3 Forestry Station of Huangnan Prefecture of Qinghai Province, 14 Regong Road, Tongren 811300, China

**Keywords:** herding proximities, reasonable management, dispersion degree, kernel density estimation, precision livestock, farming animal husbandry

## Abstract

**Simple Summary:**

We proposed a pragmatic method for quantifying the grazing density (GD) and herding proximities (HP) based on unmanned aerial vehicles. We further tested its feasibility at three typical household pastures on the Qinghai-Tibetan Plateau, China. The proposed method is ideal for studying animal behavior and determining the correlation between the distribution of pastoral livestock and resource usability.

**Abstract:**

Grazing management is one of the most widely practiced land uses globally. Quantifying the spatiotemporal distribution of livestock is critical for effective management of livestock-grassland grazing ecosystem. However, to date, there are few convincing solutions for livestock dynamic monitor and key parameters quantification under actual grazing situations. In this study, we proposed a pragmatic method for quantifying the grazing density (GD) and herding proximities (HP) based on unmanned aerial vehicles (UAVs). We further tested its feasibility at three typical household pastures on the Qinghai-Tibetan Plateau, China. We found that: (1) yak herds grazing followed a rotational grazing pattern spontaneously within the pastures, (2) Dispersion Index of yak herds varied as an M-shaped curve within one day, and it was the lowest in July and August, and (3) the average distance between the yak herd and the campsites in the cold season was significantly shorter than that in the warm season. In this study, we developed a method to characterize the dynamic GD and HP of yak herds precisely and effectively. This method is ideal for studying animal behavior and determining the correlation between the distribution of pastoral livestock and resource usability, delivering critical information for the development of grassland ecosystem and the implementation of sustainable grassland management.

## 1. Introduction

Grasslands account for about a quarter of the ground surface worldwide, and managed grazing on the natural rangeland is the most extensive and efficient utilization form [1,2]. Reasonable grazing management could improve grassland productivity, maintain and enhance biodiversity, and increase the income of local herders [3,4,5]. However, the increasing human disturbance and climate change have caused multiple ecological problems [6], such as grassland degradation, exotic species invasion, and catastrophic loss of carbon reserves [7,8,9]. It is essential to improve grazing management to regulate excessive pressure and maintain the multifunctionality of grassland ecosystems [2,10].

Grazing density (GD, also referred to as stock density) monitoring and efficiency evaluation are the key steps to improve the management of grassland [11,12]. Although, the controlled-grazing experiments have been conducted intensively in the steppe and alpine meadow ecosystems in northwestern China [13,14], while it has been acknowledged that there is big gap between real-world conditions and control experiments. Unlike the designed experiments, GD under real-world conditions have rarely been quantified because of the limitations of efficient monitor technology and available data. Some studies have focused on quantifying GD through vegetation indices and ground observation visually or by using time-lapse cameras [15,16,17], while some other researchers have attempted to quantify GD only by the remote sensing indices [1,17,18]. However, these studies usually provide rough estimates based on some specific indicators, which may not be sufficient for precise and reasonable management of grassland. Recently, Lei et al. [19] tried to produce quantitative GD based on GPS collars and kernel density estimation (KDE) methods, while the representativeness could be limited (though the temporal resolution is very high).

Developments in rangeland management have highlighted the crucial role of managing the spatial and temporal distribution of ungulate herbivory to uphold the ecosystem services and support the biodiversity conservation [20,21]. It is generally acknowledged that unlike the fence-controlled grazing, herbivores grazing freely tend to concentrate grazing in specific areas of the pasture because of the variability in both the quantity and quality of herbage [22], which could result in localized overgrazing while leaving other areas of the pasture underutilized [23]. Thus, spatiotemporal heterogeneity in pastures can pose limitations on the use of grazing as a management tool for animal husbandry production and conservation [24], and managing the distribution of herbivores is a major issue facing rangeland managers [25,26]. Some studies have been conducted to depict the spatiotemporal distribution of herbivores, e.g., Barcella et al. [27] mapped the locations of animal by direct field observation, where data points are firstly hand-painted on a paper chart and subsequently vectorized as geographic elements in GIS, and their movements are then marked by different polygons; Nakano et al. [16] monitored the herbivores by time-lapse camera within approximately 0.034 ha. These studies attempted to provide basic concepts and methods of the distribution of herbivores. However, their methods are featured with low efficiency and precision because of the visual error and complicated data processing. In recent years, the unmanned aerial vehicle (UAV) technology develops rapidly, which provides a novel tool for animal monitoring [28,29,30]. Researchers have utilized UAVs to monitor ungulate herbivores and tried to identify individuals based on the aerial images [29,31,32]. Unfortunately, few studies have revealed the dynamic herd movements and herding proximities (HP). Therefore, a new convincing and herd-based (opposed to individual-based) method is urgently needed for evaluating the spatiotemporal heterogeneity and social relation of herbivores, and UAVs provide a potential opportunity.

The Qinghai-Tibetan Plateau (QTP) is the largest and highest plateau that is known as the Third Pole, and grazing is the main management and utilization form for grasslands on the QTP [33]. The yak, featured with exceptional adaptability to the alpine environment [34,35], is one of the most important herbivores on the QTP, providing food and economical sources for the locals. Alpine grassland is the most extensive vegetation type, while 90% of them have degraded to a different extent [10,31]. The Rangeland Contract Responsibility System (RCRS) was introduced to the QTP in 1980s, leading to household pastures becoming the primary unit for management and utilization of alpine grasslands [9]. The major characteristic of this management method is that livestock graze during the day and are confined to a campsite at night, resulting in a radial grazing intensity gradient [29,36]. In this study, at three typical household pastures on the QTP, we monitored yak herds dynamically and proposed a method for calculating GD and spatiotemporal distribution of yaks. The specific objectives were to: (1) generate GD with the herd-based monitoring of UAV at the household scale; (2) characterize the spatiotemporal distribution of yak herds based on GD, and (3) analyze the variations of dispersion degree of yaks during daytime, months, and whole grazing seasons. The pasture monitoring and data analysis with the proposed methods are critical to improve our understanding of distribution pattern and social relations of livestock, which helps rationalize grazing management and promote the sustainable development of alpine grassland ecosystem.

## 2. Materials and Methods

### 2.1. Study Area

**Figure 1:**
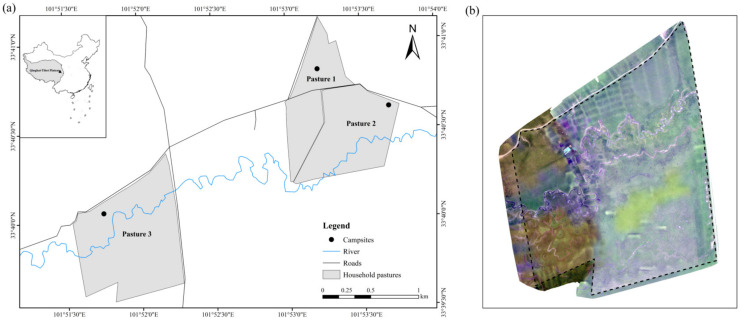
Study area on the Qinghai-Tibetan Plateaus (**a**) and the orthophotos of Pasture 3 (**b**). Lines indicate ranch borders and black circles represent campsites of each pasture.

This study was carried out at Azi Research Station, Maqu County, Gansu Province, China (Figure 1a), located in the east of the QTP (101°52′07.9″ E, 33°24′24.1″ N) with an altitude of 3540 m above sea level and featured with the typical plateau continental climate. The annual average temperature is approximately 1.1 °C, and the average annual rainfall is approximately 620 mm, mostly occurring from June to September [1,14]. The soil type is alpine meadow soil. The vegetation is typical alpine meadow dominated by monocotyledonous plants such as *Poaceae* (e.g., *Festuca ovina*, *Poa poophagorum*, etc.) and *Cyperaceae* (e.g., *Carex kansuensis* and *Kobresia graminifolia*). Some dicotyledonous species such as *Ranunculaceae*, *Asteraceae,* and *Scrophulariaceae* are also common [37].

### 2.2. Experimental Design

Three typical household pastures around Azi research station were selected (Table 1). These pastures featured with gentle topography (slope < 5°) and were managed similarly, that is, yaks move back to the campsites at night and graze freely within the pasture during the daytime, while the time of pastures transitions were different.

**Figure A1:**
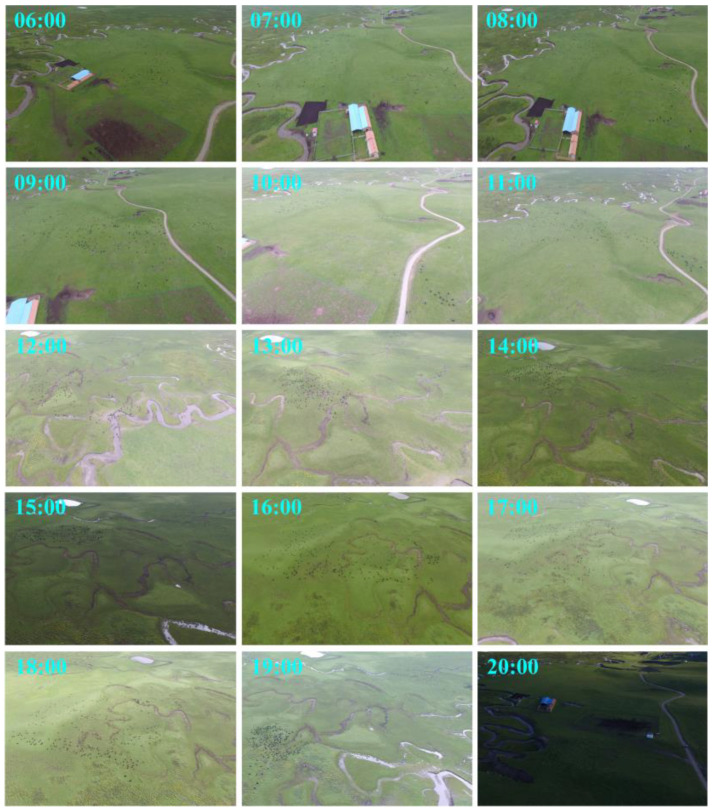
The UAV-based tracking and monitoring method.

From April to October in 2017, yak herds of three pastures were monitored (4 or more days/month). A DJI Phantom 3 Pro (equipped with a 12-megapixel RGB camera, DJI Innovation Company, China) was utilized to track and monitor the herds during the grazing periods with one (or two, occasionally) aerial photographs taken per hour for each herd, that is, the aerial photographs cover all the yak within each pasture by adjusting the flight height and shooting angle. The suitable height was 80–150 m, and the angle could be rectified by the following “Geolocations of yak extraction” processes. The aerial photographs ranging from 10 and 15 per monitoring day were taken, which depended on the actual managements of herders (Figure A1).

### 2.3. Geolocations of Yak Extraction

In order to pinpoint the geolocations of individual yaks accurately from the aerial photographs, we employed the MOSAIC flight mode to collect overlapping aerial photographs (this work was only used to obtain the base map of each pasture; overlapping was 60%, the height was 150 m, and the resolution was ~7 cm using Phantom 3 Pro in this study), which were subsequently processed to generate georeferenced orthomosaics of the pastures (e.g., Figure 1b). Briefly, FragMAP software [38] (installed and operated on Huawei M5, Shenzhen, China) was used to acquire the overlapping aerial photographs automatically, and then Pix4Dmapper software (Lausanne, Switzerland) was utilized to preprocess and generate the orthophotos of the pastures.

Yaks are commonly black or black-white, with a body length ranging from 1 to 3 m (for both calves and adults). In an RGB aerial photograph taken by UAVs, yaks are typically represented as dark or white squares, occupying tens or hundreds of pixels (depending on size, image resolution, flight height parameters, etc.). Meanwhile, grasslands are generally depicted as green (warm season) or yellow (cold season), which makes it feasible to identify yaks on the grassland based on the colors and pixel count.

**Figure 2:**
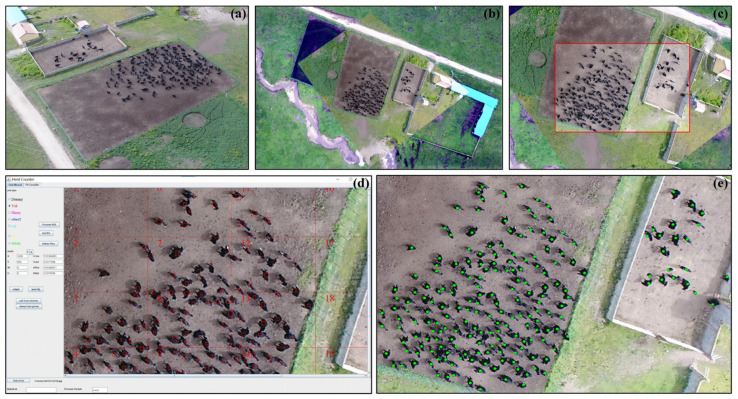
Locating of yaks. (**a**) Original aerial photograph of yak herd; (**b**) geometric correction of aerial photograph in ArcGIS; (**c**) trimmed aerial photograph of yak herd; (**d**) location of each yak by HerdCounter, which is an independently developed Java software using OpenCV library; and (**e**) verification of yak locations in ArcGIS.

To extract the geolocations of yaks efficiently, the combination of georeferencing and image recognition was employed in this study [29]: (1) aerial photograph was orthorectified according to the georeferenced orthomosaic, using at least 10 reference points in ArcGIS (Version 10.2.2); (2) the adjusted photograph was cropped using the minimum bounding rectangle (MBR) of the herd to remove invalid information; (3) spatial extent of the MBR (latitude and longitude information for the top left point and bottom right point) was saved as a TXT file; (4) geolocations of yaks were extracted automatically using HerdCounter (a Java-based software to recognize and count the number of yaks in the orthorectified images, Java version should be 1.8.0_191 or higher), and the latitude and longitude of individual yaks were saved as CSV files in text form, and (5) the yak location points were loaded and compared with the georeferenced monitoring photos (Figure 2).

### 2.4. GD Estimation

Kernel density estimation method has been widely used in the field for species distribution prediction [39,40]. This method is generally used as a mathematical tool for estimating the probability density function of the entire population based on field samples [41,42,43]. It assumes that there is a measurable event density or intensity within a given region, which can be estimated by counting the number of event points within a certain area surrounding it. In other words, by placing kernel function at each event point, the intensity in any place can be calculated by considering the influence of all kernel functions within the region. However, what we expect is not a traditional probability surface but an intensity surface of practical significance. In this study, the integral of single kernel function was redefined as the GD caused by a yak within an hour in the pasture.

Based on these foundations, we developed a method to estimate the GD at the household scale based on the KDE method:

(1) $$GD\left(x,y\right)=\frac{3\sum_{i=1}^{n}K\left(x-x_{i},y-y_{i}\right)}{sTf\pi h^{2}}A$$

where *GD* (*x*, *y*) represents the GD at point (*x*, *y*); *s* is the area of the designated pasture measured in hectares; *T* is the annual grazing time of the pasture in days; *f* is the number of times that UAV sampling is conducted to track herds in a collecting day; *h* is the bandwidth measured in meters; *n* is the number of sample points falling within the circle with the bandwidth as its radius at point (*x*, *y*); *x_i_* and *y_i_* represent the position of the *i*th data point in the projected coordinate system, which is measured in meters; *A* is an optional adjustment factor used in the monthly estimation of GD, with a default value of 1, and *K* represents a radial basis function whose value decreases as the distance between the unknown point and samples increases. In this study, the radial basis function is based on the triangle kernel function and is calculated as Formula (2) [44]:

(2) $$K\left(x-x_{i},y-y_{i}\right)=1-\frac{\left|\sqrt{{\left(x-x_{i}\right)}^{2}+{\left(y-y_{i}\right)}^{2}}\right|}{h}$$

A spatial resolution of 1 m was used in generating the GD map. Furthermore, the choice of bandwidth, a key parameter in KDE [45], was considered for the movement of individual yaks. Based on previous research [46], the hourly yak movement radius is 300 m, which was selected as the bandwidth. Actually, UAV monitoring was only carried out for 4–5 days per month, and thus an adjustment factor *A* was introduced, which is determined by the ratio of the total number of days in the month sampled to the number of monitoring days. The default value of *A* is 1 in Formula (1) for day-by-day and hour-by-hour GD estimations.

### 2.5. Yak Herds Dispersion

In this study, the threshold segmentation was utilized to calculate the Dispersion Index (DI) of yak herds. Given that yaks are social animals and tend to graze in groups, the radius of the herd is usually within the bandwidth (the farthest distance traveled in one hour). According to the principles of KDE, greater density values can be attained in positions where the point distribution is dense [47]. Therefore, by appropriately dividing the cone at a certain height, the area of the resulting segmented horizontal face can effectively represent the dispersion degree of the yak herd. In this study, DI was defined as the area of the region that exceeded the threshold after the segmentation process, and the threshold was set at 75% of the GD range.

### 2.6. Distance between Herds and Campsites

For each monitoring time, the Euclidean distances between GD peak and the campsites were measured. As yaks tend to display synchronous behavior patterns, we identified the actual locations of each herd by selecting the position with the highest hourly GD. It is worth noting that the distance measurements within the campsites were excluded in the calculations of daily averages.

### 2.7. Statistical Analysis

The calculations for GD, DI, and the distances between yak herds and campsites were performed using Python (version 3.10) in PyCharm software (version 2021.3.1 Community Edition). The normality of the data was tested using the Shapiro–Wilk test. Quadratic polynomial regression was used to explore the daily DI variation. To test for differences among average DI values across multiple monitoring months, we conducted one-way ANOVA, and due to unequal monitoring days in each month, we used the Duncan post-test to test the differences among months. Independent *t*-tests were used to estimate the significance of differences between the distances of yaks from the campsite during the cold season (CS; April, May, and October) and warm season (WS; June to August). All significance tests were two-tailed, with a significance level set at *p* < 0.05 to denote statistical significance. The statistical analyses were conducted using IBM SPSS (version 26) and GraphPad Prism (version 9.3, GraphPad Prism Software Inc., San Diego, CA, USA).

## 3. Results

### 3.1. GD Distribution within Pastures

**Figure 3:**
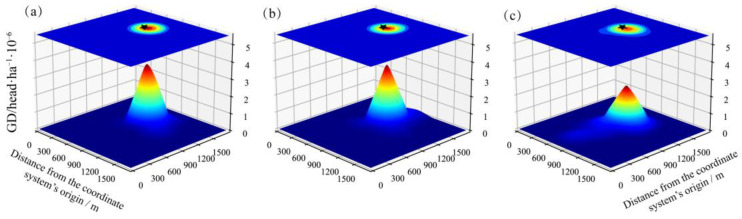
Grazing density (GD) estimation in Pasture 1 in July (**a**), August (**b**), and September (**c**) 2017. The black stars stand for the location of campsites.

**Figure A2:**
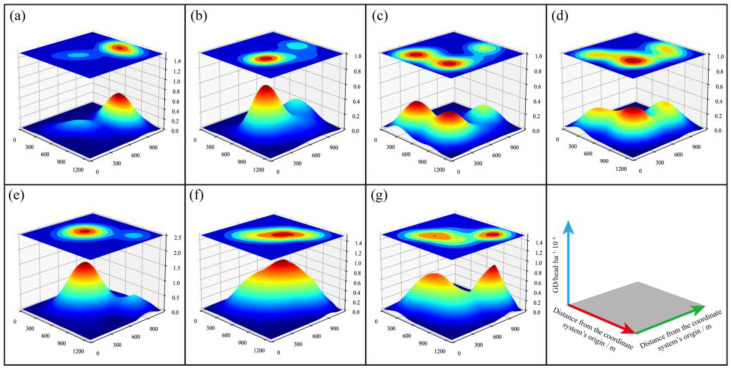
Distribution of grazing densities in Pasture 2 in 2017. (**a**–**g**) represents April–October, respectively. The pasture square boundary’s southwest corner is the coordinate system’s origin. The *x*-axis increases east and the *y*-axis north. Surface projections are shown on the top. The length units for the *x* and *y* axes are meter. The unit of grazing density is head/ha.

**Figure A3:**
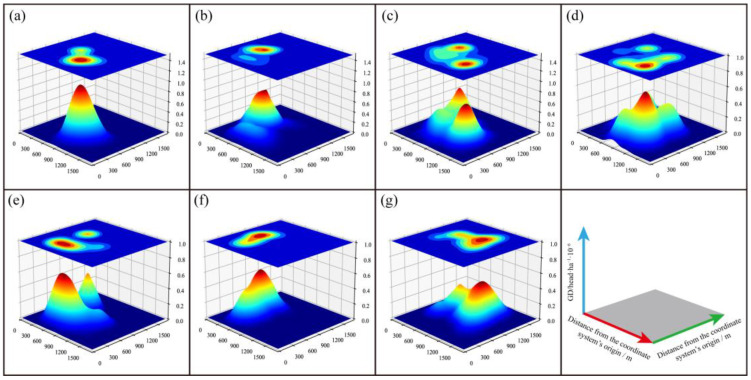
Distribution of grazing densities in Pasture 3 in 2017. (**a**–**g**) represents April–October, respectively. The pasture square boundary’s southwest corner is the coordinate system’s origin. The *x*-axis increases east and the *y*-axis north. Surface projections are shown on the top. The length units for the *x* and *y* axes are meter. The unit of grazing density is head/ha.

In Pasture 1, the GD peak was primarily located around the campsite, with a slight northward spread in July and August (Figure 3a,b), and the GD peak shifted a little to the south in September (Figure 3c). Differently, Pastures 2 and 3 were grazed since April, and exhibited a similar tendency in their GD peak movements, i.e., the GD peaks were around the campsites and then shifted to other locations. One distinct feature was that the GD peaks of different months were spread over the pastures without overlapping (Figure A2 and Figure A3).

**Figure 4:**
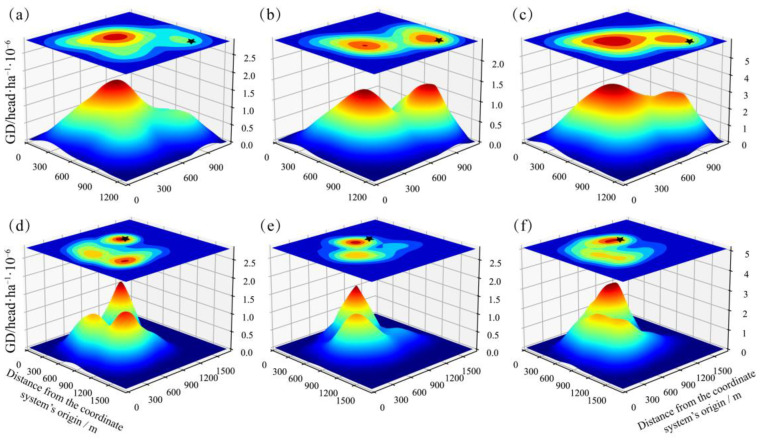
Grazing density distribution within Pastures 2 and 3 in warm (July–August) and cold season (April, May, and October), and the whole grazing period. (**a**–**c**) GD distribution in warm, cold, and whole season in Pasture 2; (**d**–**f**) GD distribution in warm, cold, and whole season in Pasture 3; the pasture’s southwest corner was set as the coordinate system’s origin, and surface projections are shown on the top. Black star is the location of the campsite. The black stars stand for the location of campsites.

**Figure 5:**
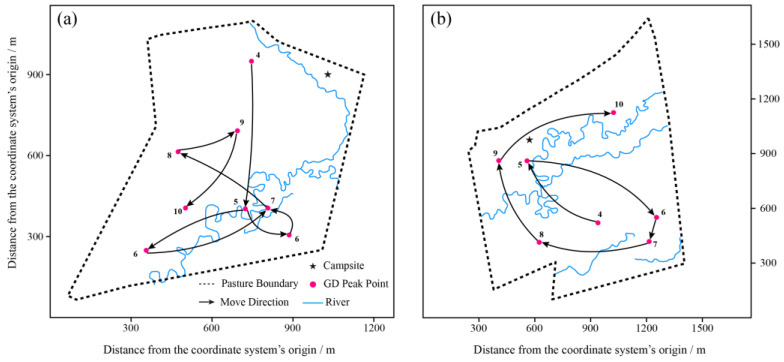
Distribution of grazing intensity peaks in Pasture 2 (**a**) and Pasture 3 (**b**) during April and October 2017. The black star stands for the positions of campsites.

In Pastures 2 and 3, yak herds were observed to spread over the pastures during both warm and cold seasons. Meanwhile, the GD was found to be heterogeneous with two or three peaks without overlap in the pastures (except the campsite areas, Figure 4 and Figure 5). Throughout the grazing period, there were two GD peaks within pastures, e.g., the campsites and the other one kept some distances from the campsites (Figure 4 and Figure 5).

### 3.2. DI of Yak Herds

**Figure 6:**
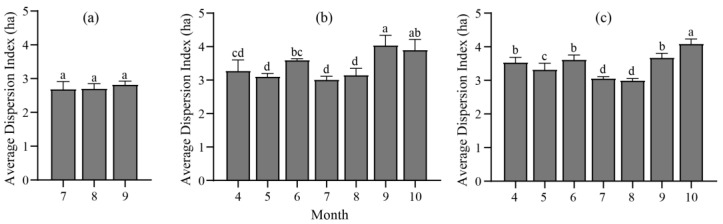
Changes in the dispersion index (DI) every month in Pasture 1 (**a**), Pasture 2 (**b**), and Pasture 3 (**c**), respectively. For each pasture, different lowercase letters indicate significant differences among monitor months at *p* < 0.05 level.

**Figure A4:**
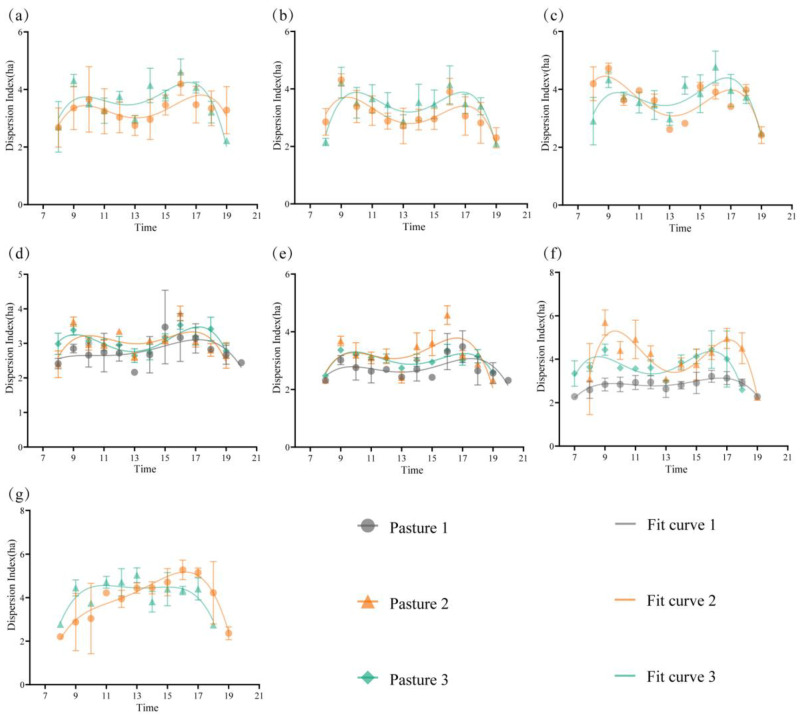
Changes in the dispersion index (DI) on daily basis. (**a**–**g**) depicts the daily fluctuation of DI for the three ranches April–October.

The daily DI followed an M-shaped curve, i.e., with peaks occurring around 9 a.m. and 4 p.m. and lower values at noon and the periods of start and end of grazing. However, this tendency was not significant for Pasture 1 (Figure A4). Regarding the monthly Dis, no significant difference was found between July and September in Pasture 1 (Figure 6a). However, for Pastures 2 and 3, the highest Dis were observed in September/October (cold season), while the lowest Dis were in July and August (warm season) (Figure 6b,c).

### 3.3. Distance from Herds to the Campsites

**Figure 7:**
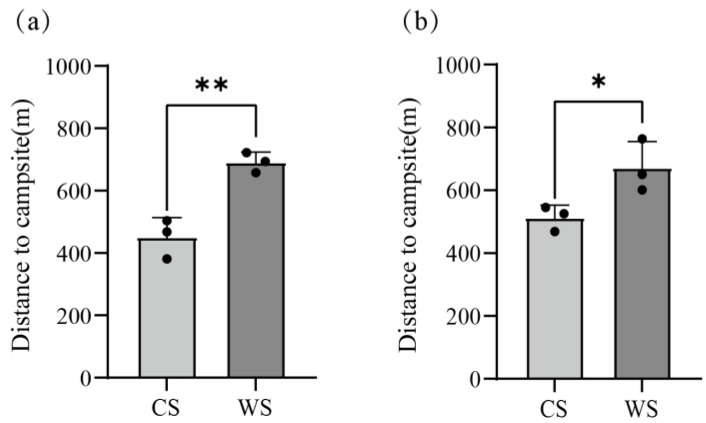
The distances to the campsite during the cold season (CS) and warm season (WS) in Pasture 2 (**a**) and Pasture 3 (**b**). * and ** represent the significance levels of *p* < 0.05 and *p* < 0.01, respectively.

The yaks grazed during July and September in Pasture 1, and the herd was distributed around the campsite (Figure 3). In Pastures 2 and 3, the yaks grazed during April and October, and the average distance between herds and campsites during the warm season was significantly longer than that during the cold season (Figure 5 and Figure 7, *p* = 0.0042 and 0.0404 for Pastures 2 and 3, respectively).

## 4. Discussion

Livestock grazing plays an important role in ecosystem conservation and socioeconomic development, especially for the alpine grassland ecosystem on the QTP [48]. Though many studies have been conducted on grazing management, the significant effects of herding behavior on foraging patterns and GD spatiotemporal heterogeneity of herbivore herds have been perceived less evidently [49], and lacking the convincing approach may be the major factor. For example, the GD measurements under real-world conditions are traditionally based on a limited number of samples (e.g., the GPS collar or monitoring some individuals visually; the samples are usually limited by financial support and labor input) and conventional statistical methods [21,29]. In addition, those methods are often hard to adequately reflect the dispersion variations that are usually ignored [50]. In this study, we propose a method for calculating the GD, DI, and the distances between herds and campsites of herbivore herds based on UAV track monitoring and the KDE algorithm. One of the advantages of the herd-based observation is that the overall distribution of the herds could be captured directly.

To our knowledge, this study is the first to document the variations in the heterogeneity of spatiotemporal distribution of yak herds, GD, and DI under real-word conditions. The GD peaks of different months and seasons were spread over the pastures without overlap, and the GDs were heterogeneous featured with two or three peaks without overlap (Figure 4 and Figure 5). The characteristics of GD imply that yaks preferred to graze in areas with lower GD in the previous period (e.g., one month interval in this study). On the one hand, it is inconsistent with the consensus that the herbivores repeatedly visit palatable patches when they are managed by season-long grazing or free grazing [21,51], while our findings also align with the conclusion of a previous study [52] that augmenting animal densities through herding (opposed to fencing) will lead to a decrease in the selectivity for palatable grass species in the savanna. Similarly, Samuels et al. [53] find that herded sheep exhibit reducing consumption of annual herbs but increasing consumption of non-succulent shrubs than the scattered sheep. The possible reason could be that herding at large spatial scales allows animals to traverse a variety of vegetation communities, preventing prolonged stays in any particular type [21]. On the other hand, the distribution of GD peaks followed a rotational grazing pattern spontaneously, which suggests that yaks can regulate their behaviors according to the changes in pasture conditions [29,46].

Animal behavior, as an outcome of intricate gene–environment interactions, serves as an intermediary factor linking anthropogenic disturbances to the fitness of animals [54]. As individual dispersal remains a central part in population dynamics and an extensively discussed topic in management programs [54], the evaluation of herding proximities has both theoretical and practical applications in the realms of grazing management and animal behavior [55,56,57]. In this study, threshold segmentation was firstly utilized to measure the DI, and it was an efficient indicator for analyzing the social relationship among herbivores. The daily DI featured with two peaks in the morning and the afternoon, and a trough at noon, which is consistent with the findings in previous studies. This M-shaped variation is believed to be associated with the foraging behaviors of ruminants, who typically forage in the morning and afternoon and engage in rumination at midday [35,58,59]. Moreover, the daily variations fluctuated modestly in July and August could be that yaks may not need to occupy a large area when food is plentiful in July and August [58,60]. Conversely, the yak herd usually kept a higher DI level in October when the forage resources become scarce, and yaks graze over larger areas to meet individual feeding requirements [35]. This behavior could be attributed to the rise in intraspecific competition for foraging with increasing herd density [61], and individuals may be stimulated by this competition to devote more time in grazing or increase intraspecific aggression. Furthermore, it could indicate that yaks significantly reduce their ruminations or resting time and increase walking time when forage quality is poor, in order to feed as much as possible to satisfy their basic energy demands [59]. DI in June and September were significantly higher than that in July and August (Figure 4), indicating that yaks tend to aggregate during the periods with higher temperature, which is inconsistent with the study of Bai et al. [3]. This result could be explained by the forage availability [60]. Therefore, it is necessary to explore the influence of feeding demands, ambient temperature, and human activities on herd dispersion and other animal behavior in the future studies [62].

Seasonal rotational grazing is the most common grazing management method on the QTP since the introduction of RCRS in 1980s [29], thus a radial grazing pattern becomes common because livestock engage in daytime grazing activities but are penned into a designated campsite overnight [36]. Different from the speculative or descriptive conclusion in the previous studies, we firstly depict the “piosphere” (an important natural phenomenon for the ecology and management of grasslands) based on actual measured values and specific algorithm (Figure 4). It provides an important potential way to study the interaction between herbivores and grassland, and one important practical application is the appropriate allocation of pastures, that is in general, herbage availability is regarded as the most important factor for pastures separation, while animal behavior is often ignored [63,64]. In the current studies, herds–campsite distance is considered as a “response to grassland condition” [29,65]. In this study, we find that the distance to the campsites in CS was significantly shorter than that in WS, which is inconsistent with the general cognition that animals would graze farther areas to obtain more forage. One possible reason could be that the tradeoff between energy accumulation and consumption [27,35,64], that is, the long period and spread over grazing within the pastures (Pasture 2 and 3), resulted in low forage availability. More important is that the herds could realize this and react accordingly. Moreover, the huge differences of GD, DI, and distance from the campsites indicate that manage pattern (especially the grazing period in this study) could affect the herd behavior significantly, implying that monitoring the herds’ behavior and further formulating reasonable manage patterns is necessary and urgent.

In this study, we introduced a herd-based method to quantify GD precisely and efficiently, taking into account the DI of the herbivores. Unlike the commonly utilized monitoring methods that estimate animal activities basing on indirect evidence only from some individuals, the UAV-based method considers data collected from the entire animal herd for analyses. However, we realize that the KDE method may underestimate GD systematically due to the boundary effects [66]. Several solutions have been proposed such as the boundary symmetry method [67], and it is necessary to explore the appropriate algorithm for the practical applications in the future studies. In addition, GD-based threshold segmentation was used to calculate DI, which facilitated the quantification of herd dispersion. Nevertheless, obtaining more data is crucial for accurate assessment and generalized conclusions, as it is crucial to note that the dynamics of animal behavior vary across spatiotemporal scales and manage patterns [25,62]. Indeed, there are some uncertainties regarding the direct application of our study’s results to other conditions (e.g., different vegetation type, animal species and composition, environmental conditions, and landforms), thus more monitored pastures and surveys may also enhance the validity of our findings. Furthermore, the herd-based geolocation (i.e., forage selection results are predominantly determined by optimizing the ratio between energy uptake and cost considerations) should be employed to determine the pastures’ allocation of a rotational grazing system on the QTP, providing insights into the reasonable utilization management of grasslands [62]—for instance, the total CS area confirmation and whether it is necessary to be separated, which would be important for survival of livestock in the harsh natural environment and sustainable development of local husbandry on the QTP. Meanwhile, the severe challenge could be how to implement the proposed method at a larger scale, and how to explore the potential influences of external factors [68,69].

In summary, the herd-based method offers distinct advantages in accurately quantifying GD and DI and also provides a foundation for further exploration of herd social relationships and potential environmental impacts. Meanwhile, the systematic underestimation of the KDE method, limited monitoring data, and herd concentration shift may impact the generalizability of results. Thus, future research could refine dispersion indices to account for partial concentration and develop more precise algorithms for determining the herd’s center of distribution.

## 5. Conclusions

The UAV-based nonintrusive monitoring approach proposed in this study is capable of depicting the GD and DI distribution of the livestock and expands the scope of livestock behavior investigation from individual-level analysis to a herd-level perspective. The spatiotemporal distribution of GDs indicates that the utilization of pasture is akin to a “rotational grazing” regime during the whole grazing season. The M-shaped variation of DI corresponds to the daily foraging behavior patterns, implying that it may fulfill a vital role in predicting dynamics and social relations and how livestock respond to environmental changes. Meanwhile, it will be necessary to address certain limitations, such as more accurate species extraction algorithms and the systematic underestimation of two-dimensional KDE. Additionally, research on the relationships between herd dynamics and biotic and abiotic factors would be extremely valuable for the sustainable development of grassland ecosystems.

## Figures and Tables

**Table 1:** Area and monitoring period of three pastures that were selected for the experiments.

Pastures	Area (ha)	~Yak Number (Head)	Monitoring Period (in 2017)
1	49.10	235	July–September
2	68.66	188	April–October
3	113.61	200	April–October

Note: the yak number are approximate values as they are always change due to calves’ birth, and adults were sold or killed by the pastoralists (or killed by wolves).

## Data Availability

Data are available from the author, Yi Sun (sunyi@ntu.edu.cn), upon request.
